# The cross-sectional area of erector spinae muscle and the liver-to-spleen ratio are associated with frailty in older patients with diabetes: a cross-sectional study

**DOI:** 10.1186/s12877-023-04347-6

**Published:** 2023-11-22

**Authors:** Motoya Sato, Yoshiaki Tamura, Yuji Murao, Fumino Yorikawa, Yuu Katsumata, So Watanabe, Shugo Zen, Remi Kodera, Kazuhito Oba, Kenji Toyoshima, Yuko Chiba, Atsushi Araki

**Affiliations:** 1 Department of Diabetes, Metabolism, and Endocrinology, Tokyo Metropolitan Institute for Geriatrics and Gerontology, 35-2 Sakaecho, Itabashi-ku, Tokyo, 173-0015 Japan; 2 The Center for Comprehensive Care and Research for Prefrailty, Tokyo Metropolitan Institute for Geriatrics and Gerontology, 35-2 Sakaecho, Itabashi-ku, Tokyo, 173-0015 Japan

**Keywords:** Frailty, Diabetes mellitus, Older adults, Erector spinae muscle, Liver-to-spleen ratio

## Abstract

**Background:**

Older patients with diabetes mellitus are more susceptible to frailty. Although some imaging markers of appendicular skeletal muscle mass obtained using dual-energy X-ray absorptiometry or computed tomography (CT) imaging can reflect frailty status, the association between imaging indices obtained by abdominal CT scans and frailty in older inpatients has not been reported.

**Methods:**

A total of 151 older inpatients with diabetes mellitus (median age, 79 years; men, 42%) who underwent abdominal CT scans close to the admission date were studied to examine the associations between abdominal CT indices and frailty. Two frailty definitions were used: the modified Cardiovascular Health Study (mCHS) criteria and Kihon Checklist (KCL) criteria. Using the imaging analysis software SYNAPSE VINCENT®, we compared the cross-sectional areas (CSA) of four truncal muscles (erector spinae, iliopsoas, rectus abdominis, and abdominal oblique muscles) and the liver-to-spleen ratio (L/S), the ratio of the CT values of the liver and spleen between frail and non-frail patients. The muscle areas that showed the strongest associations with frailty were also investigated in relation to grip strength and walking speed. Finally, multivariate binominal logistic regression analyses were performed to assess the independent associations of CSA of muscle and L/S with the prevalence of frailty.

**Results:**

The prevalence of frailty defined by the mCHS and KCL criteria was 55% and 52%, respectively. The CSA of the erector spinae muscle was most significantly associated with frailty, and was significantly smaller in both sexes of mCHS-defined frail patients and in men with KCL-defined frailty. The CSA of erector spinae muscle was also positively correlated with grip strength and walking speed. In contrast, the L/S was higher in men with KCL-defined frailty. Multivariate logistic regression analyses revealed that the CSA of the erector spinae muscle was independently associated with mCHS-defined frailty in women, and the L/S was associated with KCL-defined frailty in men.

**Conclusions:**

The CSA of erector spinae muscle and low liver fat content could be indices of frailty in older patients with diabetes.

**Supplementary Information:**

The online version contains supplementary material available at 10.1186/s12877-023-04347-6.

## Introduction

The number of older patients with diabetes mellitus has been increasing worldwide [1]. The combination of diabetes mellitus and frailty has been shown to be associated with increased mortality as well as functional disability and hospitalization [2]. Since frailty is characterized by reversibility, it can be reversed by appropriate multimodal interventions consisting of exercise, nutrition, and social participation. Thus, early detection of frailty is important in older adults with diabetes mellitus.

The most popular criteria for defining physical frailty were proposed by the Cardiovascular Health Study (CHS) [3] and included five items: unintentional weight loss, self-reported exhaustion, muscle weakness, slow walking speed, and low physical activity. Another definition of frailty is based on comprehensive geriatric assessment (CGA) tools such as the Kihon Checklist (KCL) [4] and Multidimensional Prognosis Index (MPI) [5], which include instrumental activities of daily living (IADL), mobility, oral function, cognition, depressive mood, nutrition, polypharmacy, and social isolation. Frailty evaluations based on CGA tools offer the advantage of additionally identifying the intervention domains. Moreover, KCL-defined frailty has been reported to show a good correlation with CHS-defined frailty phenotypes [4].

Several recent studies have described the use of imaging methods to assess frailty. For example, abnormalities in cerebral white matter integrity have been shown to be associated with the incidence of frailty, especially in patients with diabetes [6]. Truncal computed tomography (CT) images can provide information on body composition, including muscle and fat. Since appendicular muscle mass is one of the diagnostic parameters of sarcopenia and has been shown to be associated with functional disability, truncal muscle mass could also be a useful index for frailty or physical dysfunction. This perspective has been mainly applied in the field of surgery. Several reports have shown that the cross-sectional area (CSA) of specific truncal muscles evaluated using preoperative CT scans is associated with postoperative prognosis, including complications and mortality [7, 8].

Non-alcoholic fatty liver disease (NAFLD) causes sarcopenia through insulin resistance and inflammation [9]. “Liver-to-spleen ratio (L/S)”, the CT value in the liver divided by that in the spleen, is a good index for liver fat accumulation and may reflect insulin resistance better than the CSA of visceral fat in healthy Japanese individuals [10].

Nevertheless, no previous study has investigated the association between individual truncal muscle mass and frailty or functional disability in older patients with diabetes, and no previous reports have described a direct association between L/S and frailty. Therefore, in this study, we investigated the association between the CSAs of individual truncal muscles and frailty diagnosed by the modified CHS (mCHS) criteria and the KCL criteria in hospitalized patients with diabetes, and investigated the association between liver fat deposition and frailty.

## Methods

### Study design and participants

This cross-sectional study included 151 patients with diabetes (including 9 patients with type 1 diabetes) aged ≥ 60 years who were hospitalized for glycemic control from April 2018 to March 2021. Patients who had undergone a CT scan of the abdomen within 6 months of admission, as well as a frailty assessment during hospitalization were included. Abdominal CT scans were performed to rule out malignancy (e.g. pancreatic cancer). Participants with suspected frailty, such as slowed walking speed or exhaustion, were referred to the frailty clinic for assessment. Exclusion criteria included: a history of cerebral stroke or terminal cancer; participants whose fat/muscle borders could not be accurately recognized by the software (e.g. severely thin person); patients with significant decline in activities of daily living (ADL) or dementia who could not be assessed for frailty.

This study was conducted in accordance with the Declaration of Helsinki, and the study protocol was approved by the Ethics Review Committee of Tokyo Metropolitan Geriatric Hospital (R21-012). Since the Ethics Committee determined that written patient consent was not required, the study was conducted using an opt-out method, and consent was not obtained directly from the participants.

### CT imaging and evaluation of fat, truncal muscles, and L/S

All participants underwent abdominal CT scan in 5-mm slices without contrast. CT scans were performed based on equipment availability, not at a specific time. Abdominal muscle mass and fat mass were measured using the image analysis software SYNAPSE VINCENT® (FUJIFILM Corporation, Tokyo, Japan). This software can automatically trace visceral fat, subcutaneous fat, and the iliopsoas muscle. It has been previously reported that the cross-sectional area of visceral fat measured by CT image at umbilical level was associated with accumulation of metabolic risks [11]. Hence, the CSA of visceral and subcutaneous fat was measured at umbilical level in this study and the CT value of the fat was set from − 200 HU to -50 HU following to the manufacturer’s instructions. The CSA of iliopsoas muscle was measured using the software’s artificial intelligence engine. For the rectus abdominis, abdominal oblique, and erector spinae muscles, the muscle contours were manually traced at the lumbar L3 level and the CSAs with CT values between − 29 HU and 150 HU were defined as the muscles [12]. To obtain L/S, two > 1-cm^2^ regions of interest (ROI) on the right lobe of the liver and one > 1-cm^2^ ROI on the spleen were randomly selected and the CT values of these regions were measured. In setting the ROI, large blood vessels, intrahepatic bile ducts, and cysts were avoided. CT indices were measured by two researchers (MS and YT), with each researcher independently measuring specific aspects throughout the study.

MS was responsible for measuring subcutaneous fat, visceral fat, and iliopsoas muscle and YT measured rectus abdominis, abdominal oblique, and erector spinae muscles.

The L/S was calculated by dividing the average CT value of the two regions of the right lobe of the liver by the CT value of the region of the spleen. A low L/S indicated a high fat content in the liver, whereas a high L/S indicated a low fat content in the liver, and generally, fatty liver disease is diagnosed if L/S < 1 [13].

### Assessment of frailty

The modified versions of the mCHS and KCL criteria were used to define frailty [6]. The details of the mCHS evaluation are described in our previous report [14]. In summary, body weight loss, exhaustion, and low physical activity were assessed using questions in the KCL. Regarding low physical activity, those who answered either “No” for the question “Do you go out at least once a week?” or “Yes” for the question “Do you go out less frequently than you did last year?” were defined as positive for low physical activity. Muscle weakness was defined as a grip strength of < 28 kg in men and < 18 kg in women. Slow walking speed was defined as a speed of < 1 m/s in the 4-meter walking test. Frailty was defined by the mCHS if three of the following five criteria were met: weight loss, exhaustion, low physical activity, muscle weakness, and slow walking speed. Kihon Checklist (KCL) is a self-reporting questionnaire consisting of 25 yes/no questions (total score 25 points). Questions are designed to assess ADLs (5 points), motor function (5 points), nutritional status (2 points), oral function (3 points), cognitive function (3 points), and depression (5 points) [4]. A diagnosis of frailty by the KCL was made when the score was eight or more.

### Other clinical evaluations

The patients’ height, weight, body mass index (BMI), and blood pressure were measured on admission. Blood tests, including measurement of glycated hemoglobin A1c (HbA_1c_), albumin, lipid parameters, and estimated glomerular filtration rate, were performed at the closest time to admission and were used for the analyses. The patients underwent cognitive testing, and the Mini-Mental State Examination (MMSE) score was measured during hospitalization.

### Statistical analysis

Continuous values were compared using the Mann–Whitney test. We first compared the CSA of the muscles, fat, and L/S in abdominal CT images between men and women and between those with and without frailty according to each criterion.

We further used the chi-square test to compare the prevalence of decreased grip strength and walking speed based on the cutoff value of mCHS-defined frailty described above between those with and without decreased CSA of erector spinae muscle. The decreased CSA group was defined as those who had CSA of an erector spinae muscle below the cutoff value, which was set as the lowest tertile for each sex, and those with areas above the cutoff value constituted the non-decreased group. The correlations between the CSA of the erector spinae muscle and age, BMI, MMSE score, and blood data, and those between the L/S and these clinical indices were assessed using Spearman’s rank correlation coefficients separately by sex. Finally, multivariate binomial logistic analysis was conducted using the indices of frailty as the objective variable and the CSA of the erector spinae muscle or L/S as explanatory variables, with age, disease duration BMI, HbA_1c_, eGFR, albumin, and MMSE as covariates.

Statistical significance was set at p < 0.05. IBM SPSS Statistics ver. 26 (IBM Corp., Armonk, NY, USA) was used for statistical analysis.

## Results

### Characteristics of the patients

Patient characteristics are shown in Table 1. The study included 151 patients with a median age of 79 years (64 men [42%] and 87 women [58%]). The median BMI and HbA_1c_ level were 24.0 kg/m^2^ and 9.1%, respectively. For diabetes treatment, nine (6%) patients were prescribed diet and exercise only, 95 (62%) were taking oral hypoglycemic agents alone, 35 (23%) were taking insulin, and 22 (15%) were taking glucagon-like peptide-1 receptor agonists. On the basis of the mCHS criteria, 49.2% of men and 59.3% of women were frail, while the KCL criteria identified frailty in 44.4% of men and 57.0% of women (Supplementary Fig. 1).

**Table 1:** Clinical characteristics of patients

Age	79 [75–84]
Sex	151
Men	64(42%)
Women	87(58%)
Duration of diabetes mellitus (years)	17 [8–27]
BMI (kg/m^2^)	24.0 [21.6–27.7]
Blood pressure	
systolic blood pressure (mmHg)	133 [120–149]
diastolic blood pressure (mmHg)	70 [64–78]
HbA_1c_ (%)	9.1 [8.3–10.2]
TG (mg/dL)	116 [83–155]
LDL-C (mg/dL)	94 [77–116]
HDL-C (mg/dL)	46 [39–55]
AST (U/mL)	19 [16–27]
ALT (U/mL)	15 [12–25]
eGFR-Cre (mL/min/1.73m^2^)	58.8 [46.7–69.3]
MMSE score	27 [24–29]
SMI (kg/m^2^)	
Men	6.8 [6.2–7.4]
Women	5.7 [5.2–6.2]
Grip strength (kg)	
Men	27 [23–32]
Women	18 [15–21]
Walking speed (m/s)	1.0 [0.8–1.2]
Diabetes Treatment	
diet and exercise only	9(6%)
oral hypoglycemic agent only	95(62%)
insulin	35(23%)
Glucagon-like peptide-1 receptor agonists	22(15%)

Data are presented as median [25-75th percentile], or n (%) unless otherwise specified. Percentages do not sum to 100 because some patients were using insulin and GLP-1 receptor agonist in combination. BMI, Body Mass Index; HbA1c, glycated hemoglobin A1c; TG, Triglyceride; LDL-C, Low-Density Lipoprotein Cholesterol; HDL-C, High-Density Lipoprotein Cholesterol; AST; aspartate aminotransferase, ALT; alanine aminotransferase, eGFR, estimated Glomerular Filtration Rate; MMSE, Mini-Mental State Examination; SMI, Skeletal Mass Index

**Table 2A:** Abdominal CT indices for diabetic patients with and without mCHS-defined frailty

	Men (n = 59)				Women (n = 81)		
	non-frail group (n = 30)	frail group (n = 29)	p value		non-frail group (n = 33)	frail group (n = 48)	p value
erector spinae muscle (mm^2^)	4374 [3926, 4827]	3744 [3144, 4463]	0.010*		3627 [3251, 4072]	3290 [2807, 3747]	0.006**
abdominal oblique muscle (mm^2^)	2151 [1946, 2495]	1919 [1656, 2240]	0.030*		1588 [1389, 1907]	1527 [1325, 1769]	0.485
iliopsoas muscle (mm^2^)	1858 [1576, 2034]	1582 [1468, 2017]	0.136		1243 [1088, 1438]	1171 [873, 1356]	0.177
rectus abdominis muscle (mm^2^)	832 [740, 965]	832 [687, 985]	0.504		661 [550, 783]	600 [524, 698]	0.144
visceral fat (cm^2^)	134.2 [95.6, 177.4]	109.1 [73.6, 166.0]	0.112		112.4 [75.0, 146.5]	122.0 [84.2, 172.5]	0.569
subcutaneous fat (cm^2^)	97.8 [70.4, 150.9]	96.5 [45.8, 132.7]	0.305		175.0 [137.3, 246.0]	164.2 [101.3, 215.4]	0.308
L/S	1.16 [1.03, 1.24]	1.20 [1.14, 1.28]	0.054†		1.15 [1.04, 1.22]	1.21 [1.06, 1.31]	0.219

Data are presented as median [25-75th percentile]. mCHS, modified cardiovascular health study; L/S, CT ratio of liver and spleen. †p < 0.10. * p < 0.05. ** p < 0.01

**Table 2B:** Abdominal CT indices for diabetic patients with and without KCL-defined frailty

	Men (n = 63)				Women (n = 86)		
	non-frail group (n = 35)	frail group (n = 28)	p value		non-frail group (n = 37)	frail group (n = 49)	p value
erector spinae muscle (mm^2^)	4373 [3937, 4653]	3786 [3319, 4638]	0.036*		3504 [2989, 4017]	3477 [2865, 3787]	0.253
abdominal oblique muscle (mm^2^)	2122 [1923, 2300]	1919 [1646, 2347]	0.229		1577.5 [1312, 1862]	1556 [1325, 1762]	0.689
iliopsoas muscle (mm^2^)	1888 [1593, 2075]	1554 [1463, 1866]	0.033*		1255 [1041, 1380]	1161 [964, 1363]	0.284
rectus abdominis muscle (mm^2^)	839 [735, 973]	814 [653, 986]	0.326		665 [580, 782]	602 [483, 696]	0.041*
visceral fat (cm^2^)	126.2 [97.2, 173.2]	115.5 [60.6, 172.7]	0.34		122.0 [98.2, 157.8]	114.8 [72.3, 175.0]	0.887
subcutaneous fat (cm^2^)	97.8 [72.0, 138.5]	91.6 [50.3, 143.4]	0.68		169.7 [102.5, 241.0]	178.3 [120.7, 220.9]	0.773
L/S	1.15 [1.04, 1.22]	1.23 [1.17, 1.28]	0.008**		1.15 [1.04, 1.22]	1.22 [1.07, 1.32]	0.149

Data are presented as median [25-75th percentile]. KCL, Kihon Checklist; L/S, CT ratio of liver and spleen. * p < 0.05. ** p < 0.01

**Table 3A:** Binomial logistic regression analyses for the associations between the cross-sectional area of erector spinae muscle and mCHS-defined frailty

	Men			Women	
	Odds ratio (95%CI)	p value		Odds ratio (95%CI)	p value
Age	1.06 (0.91–1.23)	0.456		1.29 (1.10–1.51)	0.002**
Duration of diabetes mellitus	1.01 (0.95–1.07)	0.708		0.99 (0.92–1.07)	0.846
HbA_1c_	1.05 (0.67–1.65)	0.826		1.18 (0.84–1.66)	0.331
Albumin	0.85 (0.07-10.0)	0.900		0.66 (0.05–7.57)	0.736
eGFR	1.00 (0.95–1.05)	0.949		0.97 (0.93–1.01)	0.186
BMI	1.05 (0.81–1.35)	0.706		1.14 (0.99–1.32)	0.075
MMSE score	0.84 (0.69–1.02)	0.086		1.05 (0.89–1.25)	0.543
Erector spinae muscle (per 100mm^2^)	0.89 (0.78–1.01)	0.067		0.84 (0.74–0.95)	0.006**

Erector spinae muscle (mm^2^) was divided by 100 and used as a variable. mCHS, modified cardiovascular health study; HbA1c, glycated hemoglobin A1c; Body Mass Index; MMSE, Mini-Mental State Examination. ** p < 0.01. *** p < 0.001

### Associations between CT indices and the prevalence of frailty

**Fig. 1:**
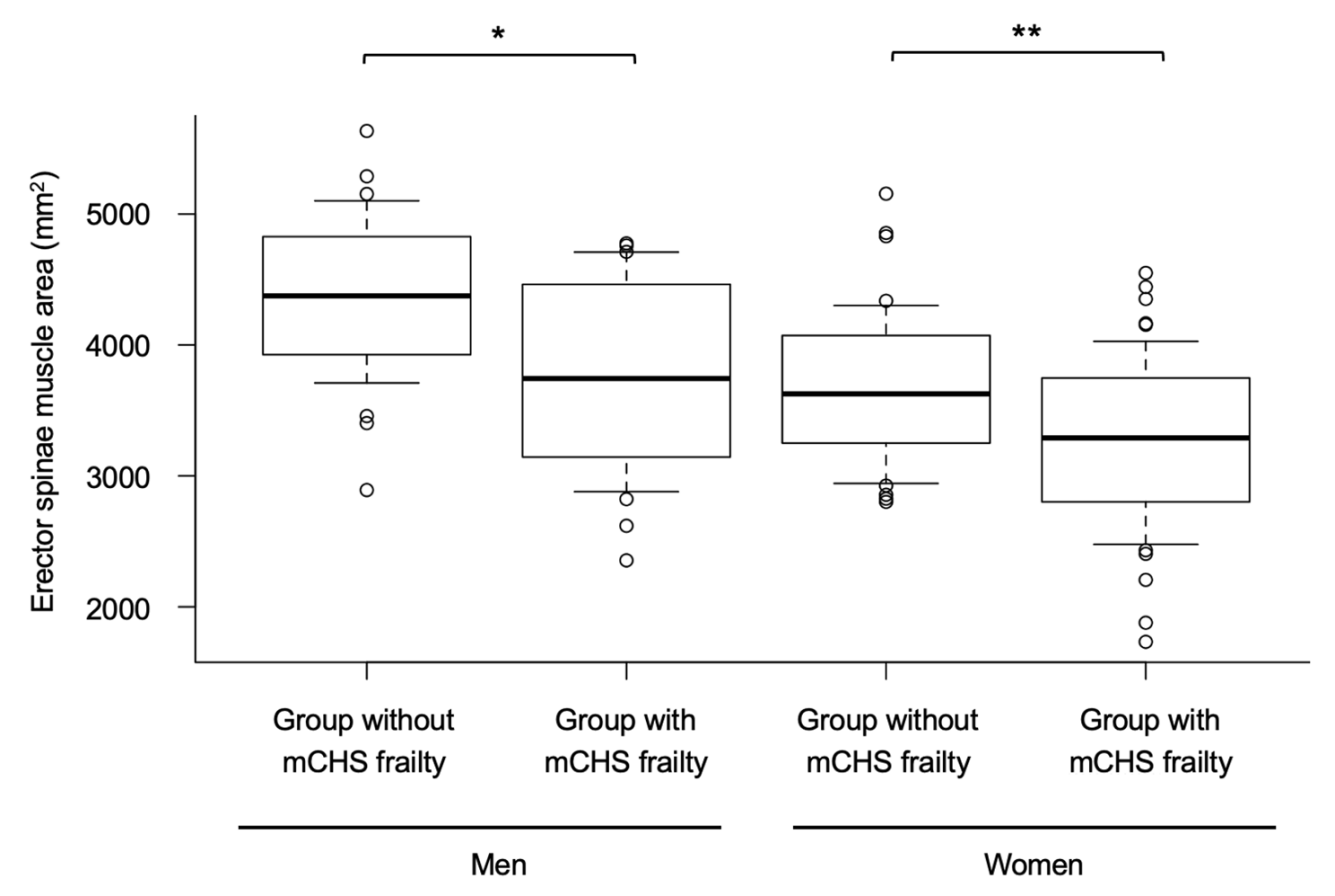
The cross-sectional area of erector spinae muscle in the groups with and without mCHS-defined frailty. The box indicates interquartile range, and the whisker indicates 10th and 90th percentile. mCHS, modified cardiovascular health study. * p < 0.05. **p < 0.01

The CT indices for mCHS-defined and KCL-defined frailty are shown in Table 2 A and 2B, respectively. In comparisons based on the presence of mCHS-defined frailty, the CSA of erector spinae were significantly lower in patients with frailty of both sexes, particularly in women (Fig. 1). Male patients with frailty showed a lower oblique muscle area and a tendency toward higher L/S. In contrast, CSA of the visceral and subcutaneous fat were not associated with mCHS-defined frailty.

In comparisons based on the presence of frailty using the KCL criteria, male patients with frailty had lower CSA of erector spinae muscle and iliopsoas muscle and a higher L/S. Female patients with frailty had a lower rectus abdominis muscle area. The CSA of visceral and subcutaneous fat were also not associated with KCL-defined frailty.

### Associations between the cross-sectional area of the erector spinae muscle and muscle strength and walking speed

Since patients with reduced CSA of erector spinae muscle showed a significantly higher prevalence of mCHS-defined frailty, we examined the association of the CSA of erector spinae muscle with grip strength and walking speed, which are components of the mCHS criteria. The results showed that patients with reduced CSA of erector spinae had decreased grip strength and walking speed than those who did not show reductions in the CSA of erector spinae (Supplementary Fig. 2).

### Associations of cross-sectional area of erector spinae and L/S with other clinical indices

We further examined the correlations of the CSA of erector spinae and L/S with other clinical parameters by Spearman’s rank correlation coefficients. The CSA of erector spinae was positively correlated with BMI and albumin level in patients of both sexes and with the MMSE score in females alone, and was negatively associated with age in females alone (Supplementary Table 1). L/S was negatively correlated with BMI and albumin in patients of both sexes, with triglyceride (TG) levels in males alone and the MMSE score in females alone, and was positively correlated with age in females alone (Supplementary Table 2).

### Binominal logistic regression analysis

Binomial logistic regression analysis with CSA of the erector spinae as the explanatory variable and frailty defined by mCHS as the objective variable showed that a low CSA of erector spinae was independently associated with the presence of mCHS-defined frailty in women after adjusting for age, disease duration, HbA_1c_ and eGFR level, BMI, albumin and MMSE score (Table 3 A), but no such association was found in men. In contrast, L/S was associated with KCL-defined frailty in men after adjusting for covariates (Table 3B); however, no such association was found in women.

## Discussion

In our study, a low CSA of erector spinae muscle was associated with mCHS-defined frailty in older patients with diabetes of both sexes in the univariate analysis and in women in the multivariate analysis. In addition, a high L/S was associated with KCL-defined frailty in males in the multivariate analysis.

Among the various abdominal muscle measurements that can be obtained by CT scans, the CSA of the erector spinae was the most associated with mCHS-defined frailty. To our knowledge, this is the first study to show an association between low trunk muscle mass and frailty in older adults with diabetes. As for the diagnostic components of mCHS-defined frailty, the CSA of the erector spinae muscle was associated with both hand grip strength and walking speed. These results suggest that the association between low trunk muscle mass and frailty may be attributable to low muscle strength as well as low physical performance.

Several reports have shown that in addition to the lower limb muscles, the trunk muscles are also involved in mobility in older adults. Masaki et al. reported that a reduction in maximal walking speed is associated with a reduction in erector spinae muscle mass in community-dwelling older adults [15]. Lim et al. reported that the erector spinae and gluteus maximus are engaged in maintaining balance during the early stance of walking in older adults [16]. Golubić et al. reported that the strength of the trunk extensor muscles, including the erector spinae muscle, is related to balance ability in healthy older women [17]. These findings indicate that a reduced CSA of erector spinae muscle can predict ADL disabilities and even mortality in older adults. Yamashita et al. reported that the CSA of erector spinae muscle evaluated by CT imaging was significantly associated with dependency in ADL at discharge in middle-aged and older patients who showed COVID-19 infection, although they did not evaluate the masses of other trunk muscles [18]. Low CSA of erector spinae muscle has also been reported to be associated with increased mortality in patients with chronic obstructive pulmonary disease [19] and lung transplantation recipients [20].

Surprisingly, CSA of visceral fat was not associated with frailty prevalence in our patients. Visceral fat deposition is known to induce insulin resistance, which is a risk factor for cardiovascular diseases and frailty. Indeed, a recent study in China reported that frail older inpatients had significantly higher waist circumference than non-frail patients [21]. Another study of Japanese community-dwelling older adults also showed that a high CSA of visceral fat was a risk factor for pre-frailty [22]. This conflicting relationship between fat accumulation and frailty can be explained by the differences in the backgrounds of the participants in the previous studies, which included non-diabetic patients, and our study, which was limited to inpatients with poor glycemic control levels. Since other risk factors for frailty, such as glycemic control and cognitive dysfunction, may play a considerable role in these patients, the influence of visceral fat accumulation on the incidence of frailty may be blunted in comparison with that in patients without diabetes.

The L/S was positively associated with KCL-defined frailty in men. We had expected a negative association since patients with nonalcoholic fatty liver disease, which presents with a low L/S, are susceptible to frailty and the disease is generally associated with insulin resistance [10] and atherosclerotic lesions [23]. However, the results were contrary to our expectations. This could also be attributed to the effects of poor glycemic control, diabetes treatment, and the age of our participants. Another possible explanation may be that the effect of undernutrition, which presents with a high L/S, on frailty may have been greater than the effect of fat accumulation in the liver in this population. In this context, it is reasonable to assume that L/S is associated only with KCL-defined frailty, which includes questions concerning body weight loss and underweight, and not with mCHS-defined frailty. Although we could not determine why the association was found exclusively in males, a sub-analysis in our previous report investigating the association between energy intake and mortality in The Japanese Elderly Intervention Trial cohort showed that low energy intake was more strongly associated with mortality in males than females [24]. Although the precise mechanism underlying this association remains unclear, it may be based on the more deteriorated malnutrition-induced outcomes in men than in women. However, since the significant association persisted even after the addition of BMI and albumin as covariates, some mechanisms other than undernutrition may be related to the association between low L/S and frailty.

Most of the results of multiple regression analyses were within our expectations: the CSA of the erector spinae and L/S were positively and negatively, respectively, associated with BMI and albumin level, indicating that both indices reflect the nutritional condition of the patients. L/S were further associated with the TG level, and liver fat content in nonalcoholic fatty liver disease has been previously reported to be associated with higher serum TG levels. Notably, both the CSA of the erector spinae and L/S were positively and negatively, respectively, associated with MMSE scores only in women. The reason for these sex differences is unclear. Since males are known to be more susceptible to atherosclerosis [25], other factors such as cerebral microvessel diseases might attenuate the influence of these indices on cognitive function in men.

The strength of this study is that we analyzed a considerable number of images of trunk muscles and L/S in older patients with diabetes who underwent CT scans during hospitalization. Moreover, we evaluated four trunk muscles separately: the erector spinae, iliopsoas, rectus abdominis, and abdominal oblique muscles.

Nevertheless, our study also had some limitations that require consideration. First, data from hospitalized patients at a single institution in Japan who underwent CT scans to rule out malignancy were used in the study. Thus, some selection bias may have existed, and our study cohort may not represent the population of community-dwelling older patients with diabetes. Also, CT scans were performed based on equipment availability, not at a specific time, which may have affected the measurement results. Second, since this was a cross-sectional study with a relatively small number of cases, the causal relationship between the imaging indices and frailty was unclear. Further longitudinal studies with a larger sample size should be planned to clarify the associations between these markers and the incidence of frailty in patients without frailty or between these markers and mortality in patients with frailty. If the CSA of erector spinae muscle is a predictor of frailty and disability, intervention studies are also required to determine whether nutrition and exercise interventions improve this muscle mass. Third, we evaluated only the areas of each trunk muscle and not their density. Recent studies have shown that the CT values of muscles, which indicate fat deposition in the muscle, are another indicator of poor prognosis [20, 26]. Sugai et al. reported that low CT values in the iliopsoas predicts major adverse cardiovascular and limb events after endovascular therapy [26]. A future study should clarify which indices of erector spine muscle, CSA or CT attenuation values, are better prediction marker for frailty in older patients with diabetes. Fourth, we were unable to evaluate certain factors in this study that could affect the prevalence of frailty, such as physical activities and social activities. Finally, we did not assess the level of insulin resistance in each patient, which could be associated with both the CSA of the erector spinae and L/S as well as the incidence of frailty. However, since our participants were patients with poorly controlled diabetes, even if the insulin or C-peptide levels were available, they might not have reflected the true levels of insulin resistance due to the so-called glucose toxicity.

## Conclusion

We found that two CT indices are associated with the prevalence of frailty in patients with diabetes: the CSA of the erector spinae was associated with mCHS-defined frailty in women and L/S was associated with KCL-defined frailty in men. Further studies are needed to investigate the effects of interventions targeting these indices, such as exercise interventions in those with low erector spinae mass or diet interventions in those with a high L/S, on the prevention of frailty or death.

**Table 3B:** Binomial logistic regression analyses for the association of L/S and KCL-defined frailty

	Men			Women	
	Odds ratio(95%CI)	p value		Odds ratio(95%CI)	p value
Age	1.02 (0.86–1.21)	0.790		1.20 (1.06–1.36)	0.004**
Duration of diabetes mellitus	0.99 (0.91–1.06)	0.706		1.05 (0.99–1.12)	0.122
HbA_1c_	0.53 (0.24–1.20)	0.130		0.68 (0.47–0.99)	0.044*
Albumin	0.068 (0.001–3.54)	0.183		1.84 (0.22–15.6)	0.575
eGFR	0.98 (0.92–1.04)	0.410		1.04 (1.00-1.08)	0.045*
BMI	1.36 (0.99–1.87)	0.059		1.01 (0.90–1.15)	0.824
MMSE score	0.75 (0.58–0.97)	0.029*		1.02 (0.88–1.19)	0.748
L/S (per 0.1)	4.95 (1.63–15.1)	0.005**		0.99 (0.77–1.28)	0.956

L/S was divided by 0.1 and used as a variable. L/S,CT ratio of liver and spleen; KCL, Kihon Checklist; HbA1c, glycated hemoglobin A1c; eGFR, estimated Glomerular Filtration Rate; BMI, Body Mass Index; MMSE, Mini-Mental State Examination. * p < 0.05. ** p < 0.01

### Electronic supplementary material

Below is the link to the electronic supplementary material.

Supplementary Material 1

## Data Availability

The datasets used and analyzed in this study are available from the corresponding author upon reasonable request.

## List of abbreviations

ADL: Activities of daily living
ALT: Alanine aminotransferase
AST: Aspartate aminotransferase
BMI: Body mass index
CGA: Comprehensive geriatric assessment
CHS: Cardiovascular Health Study
CSA: Cross-sectional area
CT: Computed tomography
eGFR: estimated Glomerular Filtration Rate
HbA_1c_: Glycated hemoglobin A1c
IADL: Instrumental activities of daily living
KCL: Kihon Checklist
L/S: Liver-to-spleen ratio
mCHS: Modified Cardiovascular Health Study
MMSE: Mini-Mental State Examination
ROI: Region of interest
TG: Triglycerides
