# Supplementary material for: The cross-sectional area of erector spinae muscle and the liver-to-spleen ratio are associated with frailty in older patients with diabetes: a cross-sectional study
Source: BMC Geriatr. 2023 Nov 22;23:765. doi: 10.1186/s12877-023-04347-6 (PMC10666293; doi:10.1186/s12877-023-04347-6)
Supplement: Supplementary file 1 — Supplementary Material 1 [file 12877_2023_4347_MOESM1_ESM.docx]

**Supplementary Table 1 Correlations between erector spinae muscle area and clinical parameters**

|  | Men | |  | Women | |
| --- | --- | --- | --- | --- | --- |
|  | correlation coefficient | p value |  | correlation coefficient | p value |
| Age | -0.237 | 0.061 |  | -0.259 | 0.016 |
| HbA_1c_ | 0.044 | 0.748 |  | -0.142 | 0.222 |
| Albumin | 0.347 | 0.005 |  | 0.283 | 0.008 |
| BMI | 0.622 | <0.001 |  | 0.286 | 0.007 |
| MMSE score | 0.086 | 0.508 |  | 0.316 | 0.003 |
| TG | 0.128 | 0.347 |  | 0.005 | 0.966 |

HbA1c, glycated hemoglobin A1c; BMI, Body Mass Index; MMSE, Mini-Mental State Examination. TG, Triglyceride.

**Supplementary Table 2 Correlations between L/S and clinical parameters**

|  | Men | |  | Women | |
| --- | --- | --- | --- | --- | --- |
|  | correlation coefficient | p value |  | correlation coefficient | p value |
| Age | 0.174 | 0.170 |  | 0.449 | <0.001 |
| HbA_1c_ | -0.114 | 0.397 |  | -0.003 | 0.979 |
| Albumin | -0.217 | 0.085 |  | -0.267 | 0.013 |
| BMI | -0.273 | 0.029 |  | -0.293 | 0.006 |
| MMSE score | -0.035 | 0.787 |  | -0.233 | 0.031 |
| TG | -0.288 | 0.030 |  | -0.202 | 0.071 |

L/S, CT ratio of liver and spleen; HbA1c, glycated hemoglobin A1c; BMI, Body Mass Index; MMSE, Mini-Mental State Examination. TG, Triglyceride.

**Supplementary Figure 1 Prevalence of mCHS, KCL frailty by sex**


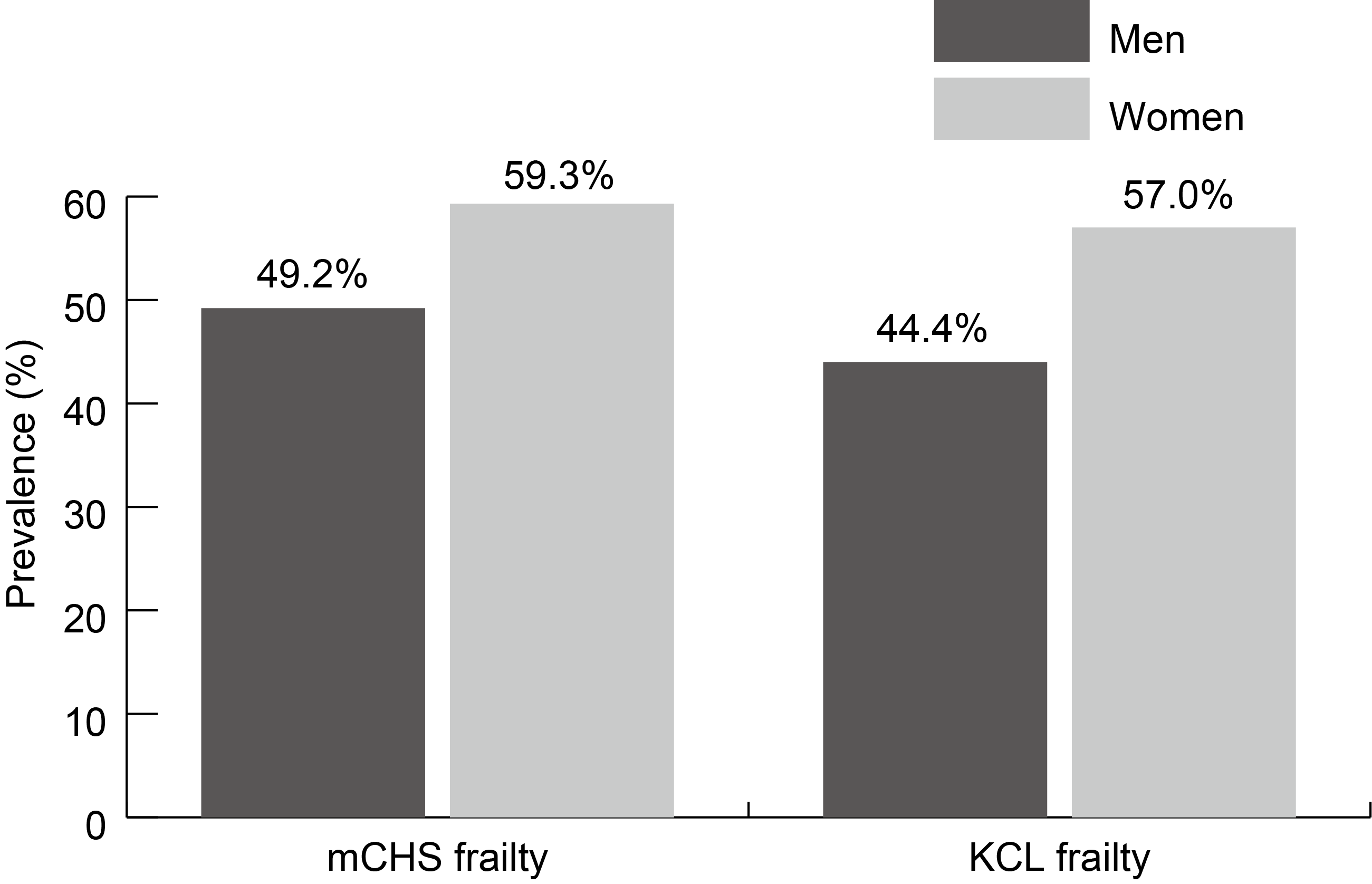


mCHS, modified cardiovascular health study; KCL, Kihon Check List

**Supplementary Figure 2 Prevalence of frailty, low grip strength, and slow walking speed**

**
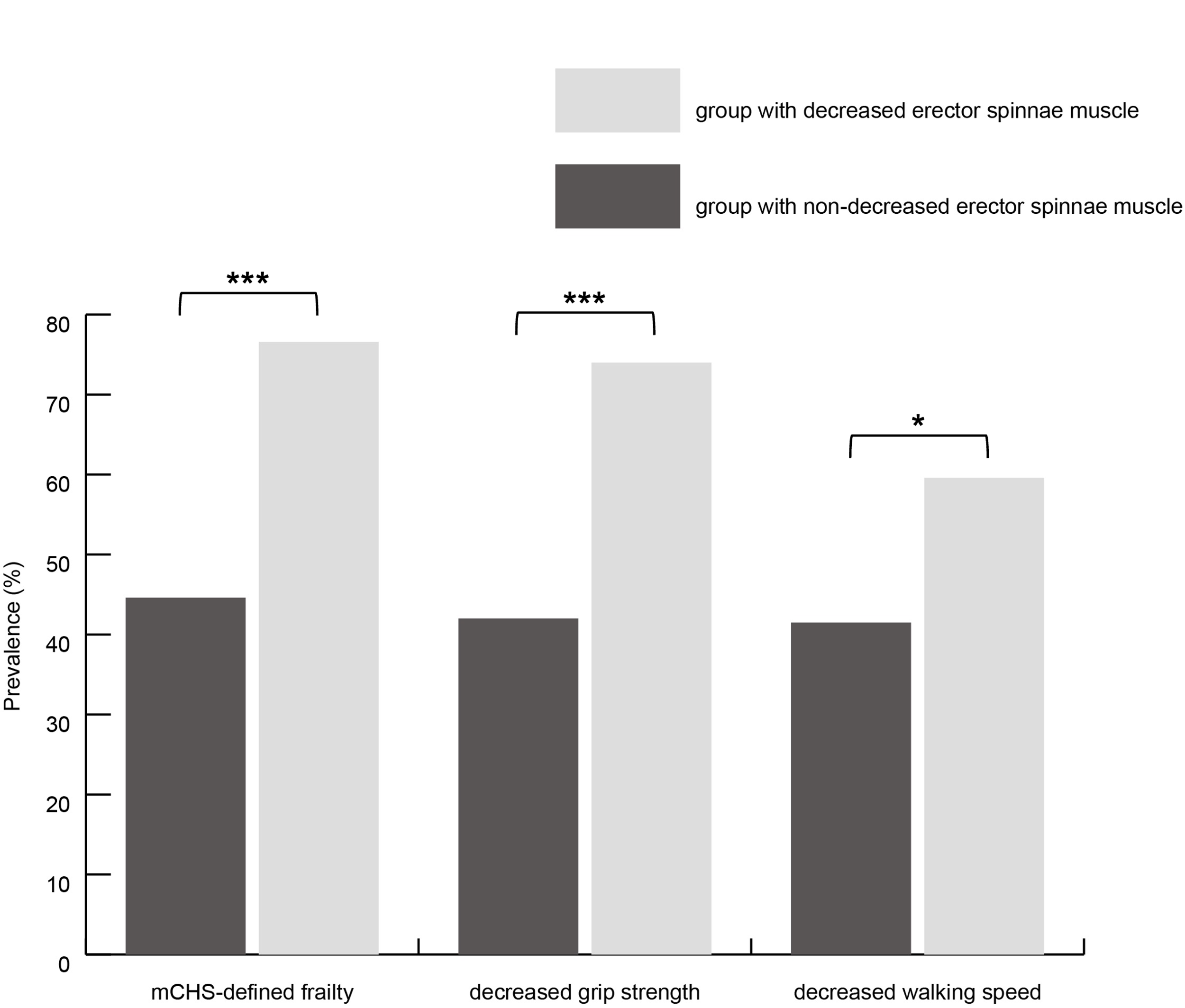
**

The χ-square test was used. * p<0.05. *** p<0.001．
